# Modulating Liposome Surface Charge for Maximized ATP Regeneration in Synthetic Nanovesicles

**DOI:** 10.1021/acssynbio.4c00487

**Published:** 2024-11-26

**Authors:** Sabina Deutschmann, Stefan Theodore Täuber, Lukas Rimle, Olivier Biner, Martin Schori, Ana-Marija Stanic, Christoph von Ballmoos

**Affiliations:** † Department of Chemistry, Biochemistry and Pharmaceutical Sciences, University of Bern, Freiestrasse 3, Bern 3012, Switzerland; ‡ Graduate School for Cellular and Biomedical Sciences, University of Bern, Bern 3012, Switzerland

**Keywords:** synthetic biology, liposomes, ionizable lipids, charge-mediated fusion, membrane protein orientation, artificial ATP production, energy conversion

## Abstract

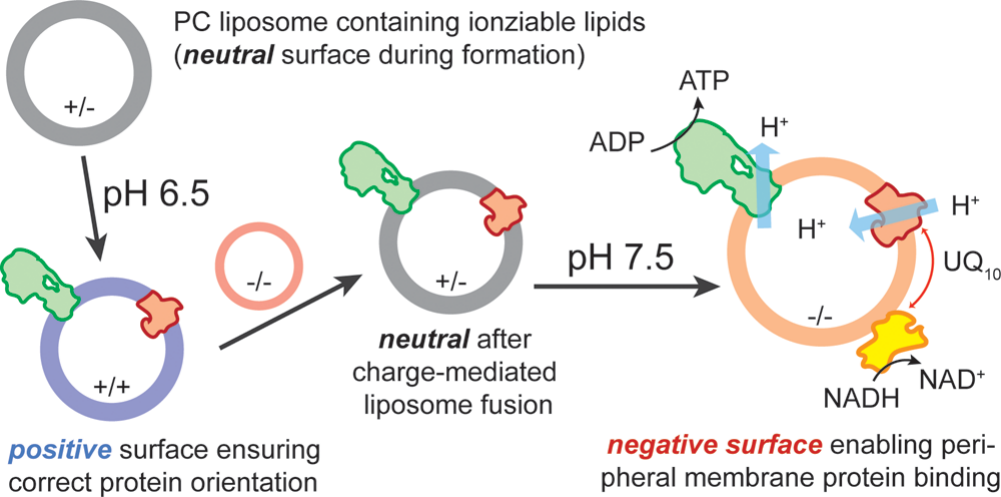

In vitro reconstructed minimal respiratory chains are powerful tools to investigate molecular interactions between the different enzyme components and how they are influenced by their environment. One such system is the coreconstitution of the terminal cytochrome *bo*_3_ oxidase and the ATP synthase from *Escherichia coli* into liposomes, where the ATP synthase activity is driven through a proton motive force (*pmf*) created by the *bo*_3_ oxidase. The proton pumping activity of the *bo*_3_ oxidase is initiated using the artificial electron mediator short-chain ubiquinone and electron source DTT. Here, we extend this system and use either complex II or NDH-2 and succinate or NADH, respectively, as electron entry points employing the natural long-chain ubiquinone Q_8_ or Q_10_. By testing different lipid compositions, we identify that negatively charged lipids are a prerequisite to allow effective NDH-2 activity. Simultaneously, negatively charged lipids decrease the overall *pmf* formation and ATP synthesis rates. We find that orientation of the *bo*_3_ oxidase in liposomal membranes is governed by electrostatic interactions between enzyme and membrane surface, where positively charged lipids yield the desired *bo*_3_ oxidase orientation but hinder reduction of the quinone pool by NDH-2. To overcome this conundrum, we exploit ionizable lipids, which are either neutral or positively charged depending on the pH value. We first coreconstituted *bo*_3_ oxidase and ATP synthase into temporarily positively charged liposomes, followed by fusion with negatively charged empty liposomes at low pH. An increase of the pH to physiological values renders these proteoliposomes overall negatively charged, making them compatible with quinone reduction via NDH-2. Using this strategy, we not only succeeded in orienting the *bo*_3_ oxidase essentially unidirectionally into liposomes but also found up to 3-fold increased ATP synthesis rates through the usage of natural, long-chain quinones in combination with the substrate NADH compared to the synthetic electron donor/mediator pair.

## Introduction

Life relies on the continuous supply of energy to fulfill a variety of tasks, such as the biosynthesis of macromolecules, transport processes, or signal transduction.^1^ The majority of this energy is converted into the universal cellular energy currency adenosine triphosphate (ATP) and is ultimately gained from either reduced-energy-rich substrates or light. ATP is primarily produced at energy converting biological membranes, where a series of respiratory complexes in mitochondria or bacteria couple electron transfer reactions to the transport of protons across the membrane. These electrogenic transport processes generate a transmembrane electrochemical proton gradient termed proton motive force (*pmf*), which serves to drive ATP production by the ATP synthase and many other transmembrane transport processes, i.e., nutrient uptake, drug efflux, ion, and pH homeostasis.

A continuous supply of ATP is also a prerequisite for bottom-up synthetic biology approaches, in which a well-defined subset of reactions is reproduced from purified components (e.g.,^2,3^). Such artificial systems have gained interest owing to their flexibility and potential to support medical processes. An important aspect is their longevity, or, in other words, sustainable use of substrates that allow a prolonged performance of the reaction. In the case of ATP, the universal energy carrier of the cell, substrate-level phosphorylation or *pmf*-driven ATP synthesis via F_1_F_O_-ATP synthase are the two most prevalent strategies.^4,5^ While the first is generally easier to accomplish, e.g., by providing creatine phosphate and creatine kinase, it suffers from product accumulation (creatine) and the requirement of constant substrate supply (PEP). The second option relies on minimal systems where a *pmf*-producing entity, e.g., a light or redox proton pump, is coreconstituted with an ATP synthase in a liposomal membrane that harbors the *pmf* to generate ATP from ADP. Here, light-driven pumps stand out with their economic mode of energization (light) and no product accumulation. However, the best-known systems with bacteriorhodopsin and, recently, proteorhodopsin are substantially less efficient than systems in which the proton pumps are redox-driven.^1^ Our group has contributed to the latter by describing the coreconstitution of the terminal *bo*_3_ oxidase with the ATP synthase from *Escherichia coli*.^2,6^ In this particular system, where the ATP synthesis rate can be monitored under steady-state conditions, proton pumping by the *bo*_3_ oxidase is initiated by the addition of water-soluble quinol ubiquinone Q_1_ in combination with an electron source (DTT).^2,6,7^ Using this setup, Nilsson et al.^6^ investigated the influence of the lipid composition on coupled ATP synthesis activity in different liposome species with varying lipid compositions and found decreased ATP synthesis if negatively charged lipids (DOPG, DOPA, and cardiolipin (CL)) were present in otherwise zwitterionic DOPC liposomes.^6^ Interestingly, the effect was dependent on the protein density and the liposome diameter, leading to the discussion of a lateral proton transfer along the membrane surface. This phenomenon attempts to describe how the equilibration of protons with the bulk solution is kinetically delayed, and protons ejected by primary proton pumps are first transferred laterally along the membrane.^8−17^ Interestingly, acid–base-driven ATP synthesis (in the absence of *bo*_3_ oxidase) was not affected by the presence of negatively charged lipids, and neither was the reconstitution efficiency of the proteins. The remaining untested variable was the relative orientation of the *bo*_3_ oxidase depending on the lipid composition, as the orientation for the ATP synthase was found to be unaffected. The orientation of the *bo*_3_ oxidase is crucial, as the substrate, reduced ubiquinol, reaches the protein via the membrane and thus activates both *bo*_3_ oxidase orientation populations, and any change in the distribution has a direct impact on the formed *pmf* and thus on ATP synthesis.^18−20^ While unidirectional orientation of membrane proteins in cells is ensured by a controlled cotranslational insertion into the membrane assisted by several factors, the orientation is much harder to control during in vitro reconstitution using detergents. In the described system, short-chain ubiquinone was used as an electron mediator shuttling electrons between the aqueous phase from the electron source (DTT) and the membrane to energize *bo*_3_ oxidase proton pumping. While this form of reduction is convenient, it comes with the drawback of increased amounts of required ubiquinone and the use of a non-natural electron source (DTT) that cannot be easily regenerated.

Here, we address this limitation by using either the membrane-embedded complex II or the peripheral NADH dehydrogenase II (NDH-2) from *E. coli* that use succinate or NADH, respectively, as electron sources to reduce the membrane-embedded native ubiquinone Q_8_ pool. We find that NDH-2 is more efficient than complex II in synthesizing ATP but requires negatively charged membrane lipids for optimal function. Using our recently described method to determine membrane protein orientation,^21^ we identified that negatively charged lipids promote the orientation of *bo*_3_ oxidase toward the less favorable right-side-out orientation and that the desired inside-out orientation is observed in the presence of a positively charged membrane that is incompatible with NDH-2 activity. To overcome this discrepancy (i.e., positive charge for *bo*_3_ oxidase orientation, negative charge for NDH-2 function), we used ionizable lipids (DODAP) that are positively charged at low pH levels and uncharged under neutral or alkaline conditions. In the first step, *bo*_3_ oxidase and ATP synthase are coreconstituted at pH 6.5, yielding a favorable oxidase orientation. Second, the lipid composition is changed by fusing empty negatively charged liposomes with the positively charged proteoliposomes, leading to uncharged proteoliposomes. Subsequent pH increases render the final proteoliposomes overall negative and thus result in a more accurate biological membrane at physiological pH that is compatible with NDH-2 activity. This strategy allows us to efficiently modulate the orientation of the *bo*_3_ oxidase without the need for protein modification and enables the use of NDH-2 as an efficient means to reduce membrane-embedded Q_8_ and energize ATP synthesis using physiological substrates.

## Results and Discussion

ATP is the major energy source in both biological systems and bottom-up synthetic biology approaches. Importantly, ATP supply has to occur continuously to retain the system out-of-equilibrium, where biological tasks such as nutrient uptake, nerve function, or anabolic reactions can be performed. Since the combination of an established transmembrane *pmf* generated by redox-coupled proton pumping coupled with an F_1_F_O_-ATP synthase is found in all organisms, we aim to efficiently retain this essential process in a minimal system with reduced complexity.

### Three-Component Artificial *E. Coli* Respiratory Chain

**Figure 1:**
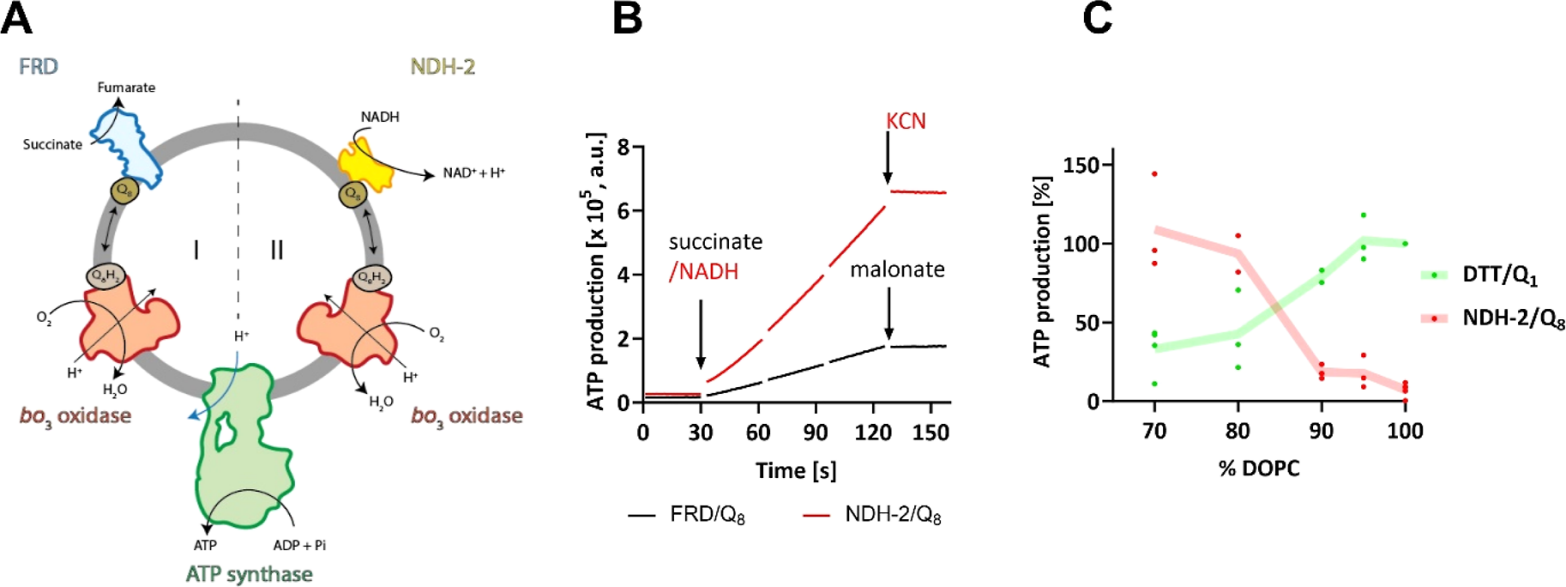
(A) Bottom-up approaches of artificial respiratory chains using*E. coli* enzymes. For approach I, FRD, *bo*_3_ oxidase, and ATP synthase are coreconstituted into liposomes containing electron mediator ubiquinone Q_8_. The addition of succinate leads to Q_8_ reduction by FRD, followed by the reoxidation of Q_8_H_2_ and simultaneous proton translocation through *bo*_3_ oxidase. The so-generated *pmf* is used by F_1_F_O_-ATP synthase to produce ATP, which in turn is detected via luminescence. In an alternative approach II, Q_8_ is reduced upon NADH addition by the peripheral membrane protein NDH-2, which can be added to proteoliposomes during measurements. (B) Approach I (black trace)–ATP synthesis was initiated by adding 1 mM succinate and inhibited by 400 μM of FRD inhibitor malonate. Approach II (red trace)-to start ATP synthesis, 300–500 nM NDH-2 and 200 μM NADH were added, and ATP production was monitored. The reaction was stopped by *bo*_3_ oxidase inhibitor KCN. (C) Coupled ATP synthesis rate initiated either by NDH-2/NADH or Q_1_/DTT in DOPC liposomes containing varying amounts of DOPG. For Q_1_/DTT-induced ATP synthesis, 20 μM Q_1_ and 4 mM DTT were used to start the reaction, while NDH-2/NADH-induced ATP synthesis was measured, as described in (B). Rates were normalized to 100% DOPC (Q_1_/DTT induced).

We showed that liposomes containing coreconstituted *bo*_3_ oxidase and ATP synthase (e.g.^2^) exhibited excellent ATP synthesis rate^1^ with electrons required for *bo*_3_ oxidase activity supplied from the outside by the addition of DTT and the water-soluble ubiquinone analogue Q_1_. In many bacteria, including *E. coli*, the host organism of oxidase and ATP synthase used in our study, the entry points are different dehydrogenases which extract electrons from soluble reduction equivalents produced by the oxidative breakdown of nutrients (e.g., NADH, succinate) and deliver them to the membrane-embedded ubiquinone Q_8_ pool. In a first set of experiments, we thus aimed to mimic the natural scenario with the purified enzymes of quinone-fumarate oxidoreductase (FRD) or the nonproton pumping NADH dehydrogenase NDH-2 (Figure 1A). Suitable liposomes matching the natural conditions were produced using *E. coli* polar lipid extract mixed with ubiquinone Q_8_ in chloroform, which was subsequently evaporated, and liposomes were formed using the thin lipid layer rehydration technique.^22^

In the first scenario (Figure 1A,I), purified FRD, *bo*_3_ oxidase, and ATP synthase were mixed with Q_8_-containing liposomes pretreated with 0.6% Na-cholate to enable membrane protein reconstitution according to Rigaud et al.^23^ and Nordlund et al.^24^ Na-cholate forms small micelles that can be conveniently removed by using a short gel filtration column. In vivo, FRD catalyzes the oxidation of succinate to fumarate and shuttles the electrons via the Fe/S cluster to the membrane-embedded ubi- or menaquinone. The reaction was thus started by addition of succinate, and ATP synthesis was followed in situ using the luciferin/luciferase system that emits luminescence in relation to the ATP content. As depicted in Figure 1B (black trace), ATP synthesis was detected immediately after succinate addition, indicating FRD-catalyzed ubiquinone Q_8_ reduction, reoxidation of Q_8_H_2_ by the proton translocating *bo*_3_ oxidase and simultaneous *pmf* generation, and finally ATP synthesis by the F_1_F_O_-ATP synthase. The reaction was sensitive to the complex II inhibitor malonate. Titration of succinate and ubiquinone Q_8_ dependence is presented in Figure S1A,B.

In the second scenario (Figure 1A, II), FRD was omitted from the reconstitution mix, and as NDH-2 is a peripheral membrane protein, it was added directly to the proteoliposome suspension during the measurement. As depicted in Figure 1B (red trace), immediate steady state ATP synthesis was measured when both NDH-2 and NADH were present (titration of the relevant parameters quinone Q_8_, NADH, and added NDH-2 are shown in Figure S1B,C,D). In comparison to the FRD liposomes, significantly higher ATP synthesis rates were obtained, with the numbers of *bo*_3_ oxidase and ATP synthase per liposome being similar in the two experiments, indicating that the reduction of the membrane-embedded quinone pool is the rate limiting step. Indeed, if we titrated the amount of NDH-2 to the liposome at a constant NADH concentration, we found a strong correlation between NDH-2 and ATP synthesis concentration with an apparent *K*_m_∼ of 50 nM (Figure S1D). Taken together, the data favor use of NDH-2 over FRD, as NDH-2 is easier to purify and obtained in higher yields than FRD and lacks the requirement of reconstitution and can be added in situ during measurements. In addition, the NADH/ubiquinone redox couple (*E*′_0_ = −320/45 mV) has a thermodynamic advantage over the succinate/ubiquinone reaction (*E*′_0_ = 31/45 mV) and is more suitable for synthetic biology applications. We compared our apparent *K*_m_ values for succinate, Q_8_, and NADH (Figure S1) to previously reported *K*_m_ values and found that our data closely matched literature values,^2,25,26^ indicating that the synthetic respiratory systems mimic the physiological situation well.

### NDH-2 Requires Negatively Charged Lipids for Optimal Activity

In the following, the more efficient NDH-2 setup was tested with different lipid compositions. In experiments using DTT and Q_1_ as the electron source and mediator, respectively, we had found previously that pure zwitterionic PC liposomes performed significantly better than mixtures containing negatively charged PG or cardiolipin, and this scenario was reproduced here (Figure 1C, green trace). In a similar experiment with NDH-2 and membrane-embedded Q_8_, however, essentially no ATP synthesis was found under pure PC conditions (Figure 1C, red trace), but instead only appeared in the presence of ≥20% PG, which is close to the physiological membrane composition of *E. coli*. For better readability, DOPG and DOPS liposomes are termed PG and PS in the following.

**Figure 2:**
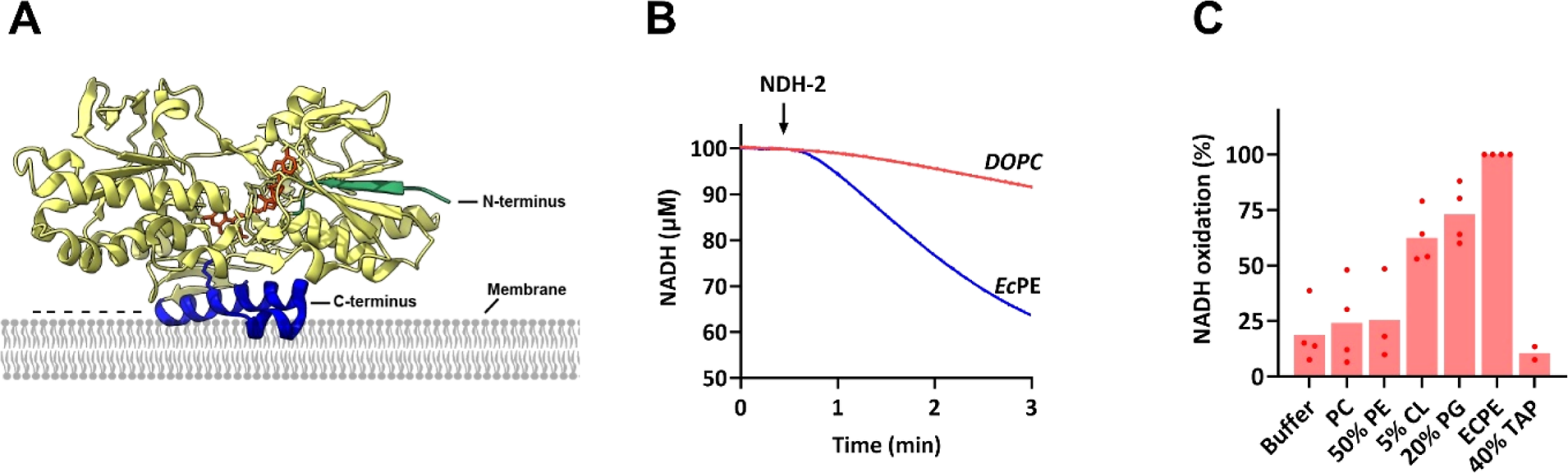
(A) Homology model of *E. coli* peripheral membrane protein NDH-2 (PDB access: 6BDO, from *C. thermarum*). Amino acids in the N-terminal domain are colored green. The C-terminus is depicted in blue, while the FAD cofactor is highlighted in orange. Interaction of the C-terminal helices to the negatively charged membrane (gray) is indicated. (B) NADH/quinone oxidoreductase activity measurement of NDH-2. Absorption of NADH is monitored at 340 nm. After reaching a baseline of buffer (20 mM HEPES pH 7.4, 200 mM NaCl, 20 mM KCl) containing 100 μM NADH, 1 mg/mL liposomes, and 100 μM Q_2_, NADH oxidation is initiated by the addition of 5-10 nM NDH-2. (C) Lipid-dependent NDH-2 activity. NADH oxidation activity of NDH-2 was measured in the presence or absence (buffer) of different liposomes (1 mg/mL), as described for (B). To adjust for different specific activities of protein preparations, measurements from different NDH-2 batches have been normalized to activity with ECPE (100%). CL: cardiolipin and ECPE: *E. coli* polar extract.

NDH-2 of *E. coli* is a 45 kDa peripheral membrane protein that catalyzes the nonelectrogenic reduction of membrane-embedded ubiquinone by NADH.^25^ It has been proposed that the protein docks to the cytoplasmic membrane via its two C-terminal amphipathic helices, which are rich in positive and hydrophobic amino acids^27,28^ (Figure 2A). Truncation of the C-terminal domain leads to a cytoplasmic protein.^28^ Typically, the protein contains a noncovalently bound FAD that acts as an NADH binding site that does not overlap with the predicted ubiquinone binding site, indicating electron tunneling between the two redox active groups.^29^ To better understand the interaction of the protein with the membrane, we spectroscopically followed the NADH oxidation activity of NDH-2 in the presence of DOPC or *E. coli* polar extract liposomes (1 mg/mL), using short chain ubiquinone Q_2_ as an electron acceptor, and solubilized *bo*_3_ oxidase was added to keep the quinone pool oxidized (Figure 2B).

As seen in Figure 2B, NADH oxidation is strongly accelerated under Michaelis–Menten conditions in the presence of *E. coli* polar extract liposomes (1 mg/mL) that contain negatively charged lipids when compared with the scenario with pure DOPC liposomes.

Next, we measured the same activity in the presence of liposomes (1 mg/mL) of different controlled lipid compositions, and our results show that NADH/ubiquinone oxidoreductase activity is indeed lipid-dependent (Figure 2C), with higher activity in the presence of net negatively charged liposomes (ECPE, PG, and CL) than in the presence of uncharged DOPC liposomes or in the absence of any liposomes (buffer). Dependent on the preparation of NDH-2, the effect of the negatively charged lipids varied measurably, however, without affecting the overall picture. The difference might arise from different levels of copurified lipids and is discussed later. The lowest activity was measured in the presence of liposomes containing positively charged DOTAP (1,2-dioleoyl-3-trimethylammonium-propane) lipids that seem to inhibit NDH-2 activity. This latter finding indicates that NDH-2 interacts with all liposomes independent of their surface charge but that only the negatively charged lipids help to mediate efficient electron transfer from FAD to the ubiquinone molecule, e.g., by a conformational change induced by electrostatic interactions that positions the two redox groups ideally for electron transfer. Taken together, the data strongly indicate that negatively charged lipids are required for NDH-2 activity, thus explaining the ATP synthesis results from Figure 1C by suboptimal reduction of the membrane-embedded quinone pool in DOPC vesicles.

### Negatively Charged Lipids Favor Undesirable *bo*_3_ Oxidase Orientation

**Figure 3:**
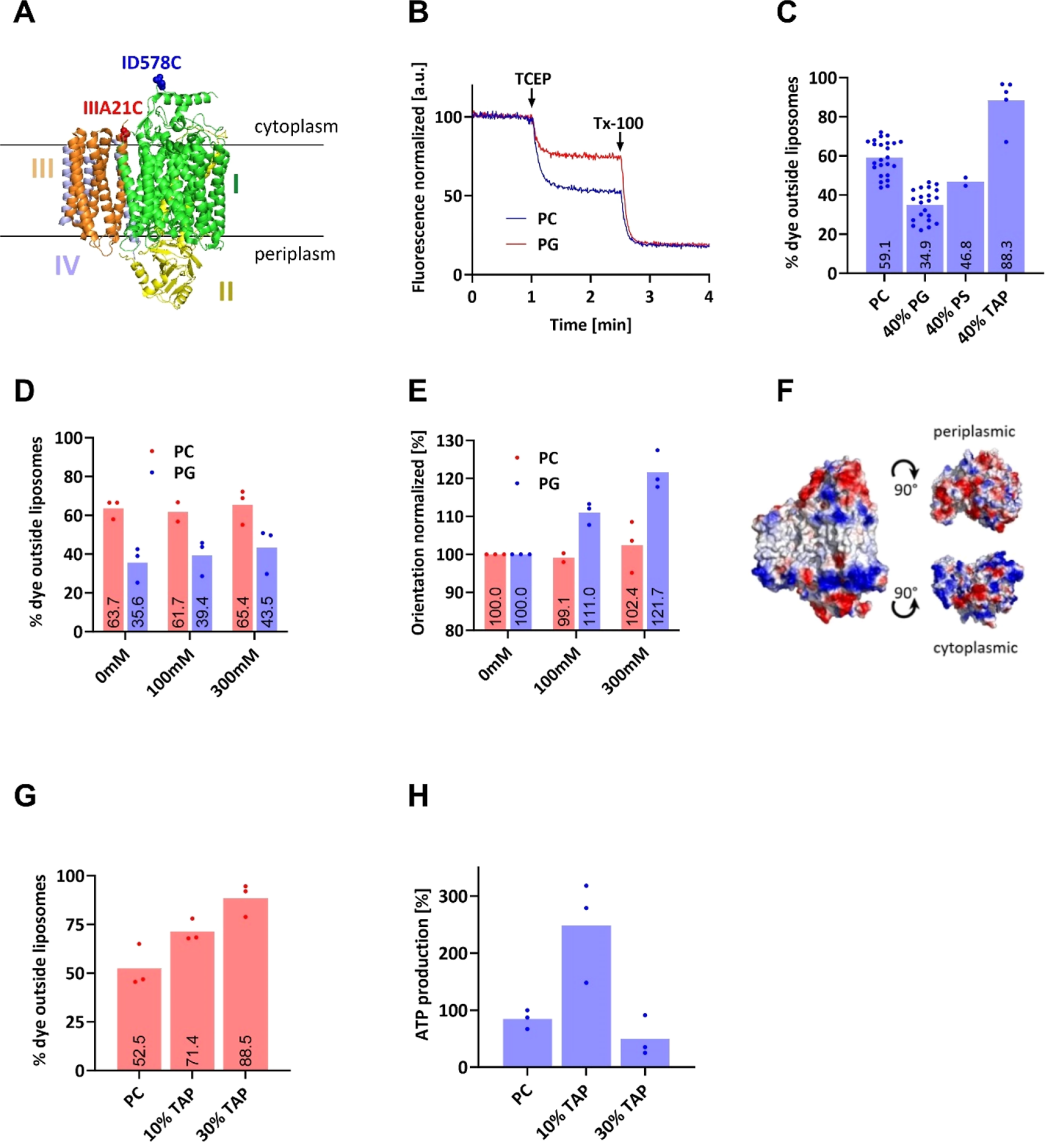
(A) Structure of *bo*_3_ oxidase (PDB access: 6WTI). Single-cysteine mutants used for orientation determination are depicted in spheres. Cysteines were located at the cytoplasmic side (ID578C or IIIA21C). (B) TCEP-based orientation determination of *bo*_3_ oxidase. Site-specifically DY647P1-labeled *bo*_3_ oxidase mutants are reconstituted in liposomes. To determine the orientation, fluorescence was monitored, and fluorophores located on the outside of liposomes are quenched in a first step by 14 mM TCEP. Full quench was achieved in a second step after adding 0.05% Triton X-100. To calculate the orientation, the first quench was set in relation to the full quench. Liposomes were composed of either only PC or 6:4 PC/PG. (C) *bo*_3_ oxidase orientation in different liposomes. Different liposomes (10 mg/mL) were partially solubilized by 0.4% sodium cholate, and *bo*_3_ oxidase was added. After detergent removal by gel filtration, liposomes were pelleted by ultracentrifugation, and orientation was determined via the TCEP-based assay. Liposomes were composed of either 100% PC, or of 60% PC and either 40% PG, 40% PS, or 40% TAP. (D,E) Orientation of *bo*_3_ oxidase after reconstitution in the presence or absence of salt. DY647P1-labeled *bo*_3_ oxidase-IIIA21C was reconstituted in absence or in the presence of 100 mM/300 mM NaCl either in pure PC liposomes (PC) or in 4:6 PG/PC (PG). Orientation was determined via TCEP-based assay and depicted in bar plots either as a fraction of inside-out orientation (D) or normalized to the orientation in the absence of salt (E). (F) Surface charge distribution of *bo*_3_ oxidase with side view (left) and top and bottom view (right), respectively. Positively charged and negatively charged areas are colored in blue and red, respectively (drawn with PyMOL with PDB access 6WTI). (G) Orientation of *bo*_3_ oxidase after coreconstitution with ATP synthase into liposomes containing TAP lipids. (H) Relative ATP synthesis rates of proteoliposomes of (G) energized with DTT/Q_1_.

Previously, Nilsson et al.^6^ found that the presence of negatively charged lipids decreases the ATP synthesis efficiency in a *bo*_3_ oxidase and ATP synthase coreconstitution experiment, and the highest activity was found in pure DOPC liposomes. A possible explanation that could not be investigated in the previous work is a lipid-dependent orientation of *pmf*-generating *bo*_3_ oxidase during the reconstitution process. As both orientation populations are activated by quinol in the membrane part of the enzyme, a shift in the orientation distribution has a direct effect on *pmf* strength and thus ATP synthesis rate.^30,31^ We recently described methodology to determine the orientation of membrane proteins in liposomes independent of their activities by side-specific quenching of a protein-attached fluorophore.^21^ Here, we used this approach expressing and purifying single-cysteine mutants D578C on subunit I or A21C on subunit III on the N-side of *bo*_3_ oxidase (Figure 3A) that were both labeled specifically with cyanine fluorophore (DY647P1) via maleimide chemistry (Figure S2). Orientation was determined by sequential quenching of the external and total fluorescence by membrane impermeable TCEP before and after solubilization of the liposomes with Triton X-100, respectively (Figure 3B). Representative raw traces of *bo*_3_ oxidase (ID578C-labeled) reconstituted in pure PC or 6:4 PC: PG liposomes are shown in Figure 3B, indicating a pronounced difference in orientation. In the PC/PG liposomes, only ∼30% of the fluorescence is quenched upon addition of TCEP, indicating that the majority of oxidases are reconstituted with their cytoplasmic side on the inside of liposomes, i.e., a right-side-out orientation. This is in contrast to pure PC liposomes, where ∼60% are oriented in the inside-out orientation that is suitable for *pmf* generation needed by the ATP synthase, explaining the increased ATP synthesis activity observed in pure PC liposomes. The results of all measurements are summarized in Figure 3C, with every point indicating a separate reconstitution experiment. While the orientation of *bo*_3_ oxidase in pure PC liposomes is around 60% inside-out, only 35% inside-out orientation is found in liposomes containing 40% PG lipids, in good agreement with other methods that found a 70% right-side-out orientation in polar *E. coli* extract.^32^ The effect of negatively charged lipids was corroborated in liposomes containing PS as negatively charged lipids, although the effect was slightly less pronounced (47%, Figure 3C). In contrast, in the presence of positively charged DOTAP lipids, an inside-out orientation of the *bo*_3_ oxidase up to ∼90% was achieved. Taken together, the data strongly indicate that the orientation of *bo*_3_ oxidase is affected by electrostatic interactions between the protein and membrane during the reconstitution process. Consequently, the presence of a high salt concentration should weaken these interactions and influence the orientation distribution. Cytochrome *bo*_3_ oxidase was reconstituted either in the absence or presence of salt (100 and 300 mM NaCl), and the orientation was analyzed by the TCEP assay. As depicted in Figure 3D,E, the presence of salt led to ∼20% relative increase in the fraction of inside-out orientated enzymes in negatively charged liposomes (PG), while a similar salt effect was not observed in zwitterionic PC liposomes. This finding can be rationalized based on the surface charge distribution of the *bo*_3_ oxidase of *E. coli* (Figure 3F). While the enzyme has almost symmetric extramembranous protrusions of similar size (which is another determinant of protein orientation^33^), the cytoplasmic side is considerably more positively charged than the periplasmic side, which shows an overall negative surface charge. If electrostatic interactions guide the contact between solubilized protein and detergent-destabilized liposomes during reconstitution, a right-side-out orientation (with the cytoplasmic side on the inside of negatively charged liposomes) would be preferred, in agreement with observed measurements. A similar mechanism has been proposed for the reconstitution of proteorhodopsin,^18^ leading to a preferable right-side-out orientation. For other enzymes with a large cytoplasmic soluble domain, such as the ATP synthase or complex I, the presence of the large cytoplasmic extramembranous moiety that is unable to cross the membrane dominates the orientation, and the proteins are preferably found in their inside-out orientation.^3,30^

Up to now, we investigated the impact of lipids on the *bo*_3_ oxidase orientation in single enzyme reconstitutions only. Very much to our surprise, we found that the presence of ATP synthase had a significant and highly reproducible effect on the *bo*_3_ oxidase orientation. While the general trend that the proportion of inside-out oriented *bo*_3_ oxidase is higher in PC than in negatively charged PC/PG or PC/CL liposomes is retained, we found that the presence of ATP synthase universally increases the yield of inside-out oriented *bo*_3_ oxidase by ∼20% in liposomes, independent of the liposome composition (Figure S3). The reason for this effect is not clear and will be discussed in the concluding remarks section.

### Ionizable Lipids Allow Temporal Modulation of Liposomal Surface Charge

Based on the findings above, reconstitution of the *bo*_3_ oxidase in the presence of positively charged lipids favors the desired inside-out orientation required for successful ATP synthesis. Assuming that all *bo*_3_ oxidases are activated independent of their orientation by membrane-bound ubiquinone, a shift in the orientation distribution should have a direct effect on the final *pmf* and thus the efficiency of ATP synthesis. We tested this hypothesis by coreconstituting the ATP synthase and the *bo*_3_ oxidase in the presence of 10% or 30% positively charged lipids DOTAP (Figure 3G,H) or EPC (1,2-dioleoyl-*sn*-glycero-3-ethylphosphocholine) (Figure S4A,B). Using fluorescently labeled *bo*_3_ oxidase, both the orientation and the ATP synthesis rates were determined from the same proteoliposomes. Compared to pure DOPC liposomes, orientation was increased in the presence of both lipids already at 10% (70–75%) and reached 90–100% at 30% positively charged lipids. A clear increase in ATP synthesis rate (powered by DTT/Q_1_) was also observed in the presence of 10% positive lipids (∼2–3 fold), while ATP synthesis was lower (DOTAP) or essentially absent (EPC) in the presence of 30% cationic lipids. This indicates that higher concentrations of positively charged lipids in liposomes are incompatible with enzyme function, likely due to a changed distribution of protons close to the membrane surface. A negative effect on F_1_F_O_-activity but not on its orientation in positively charged SUVs has been described.^34^

**Figure 4:**
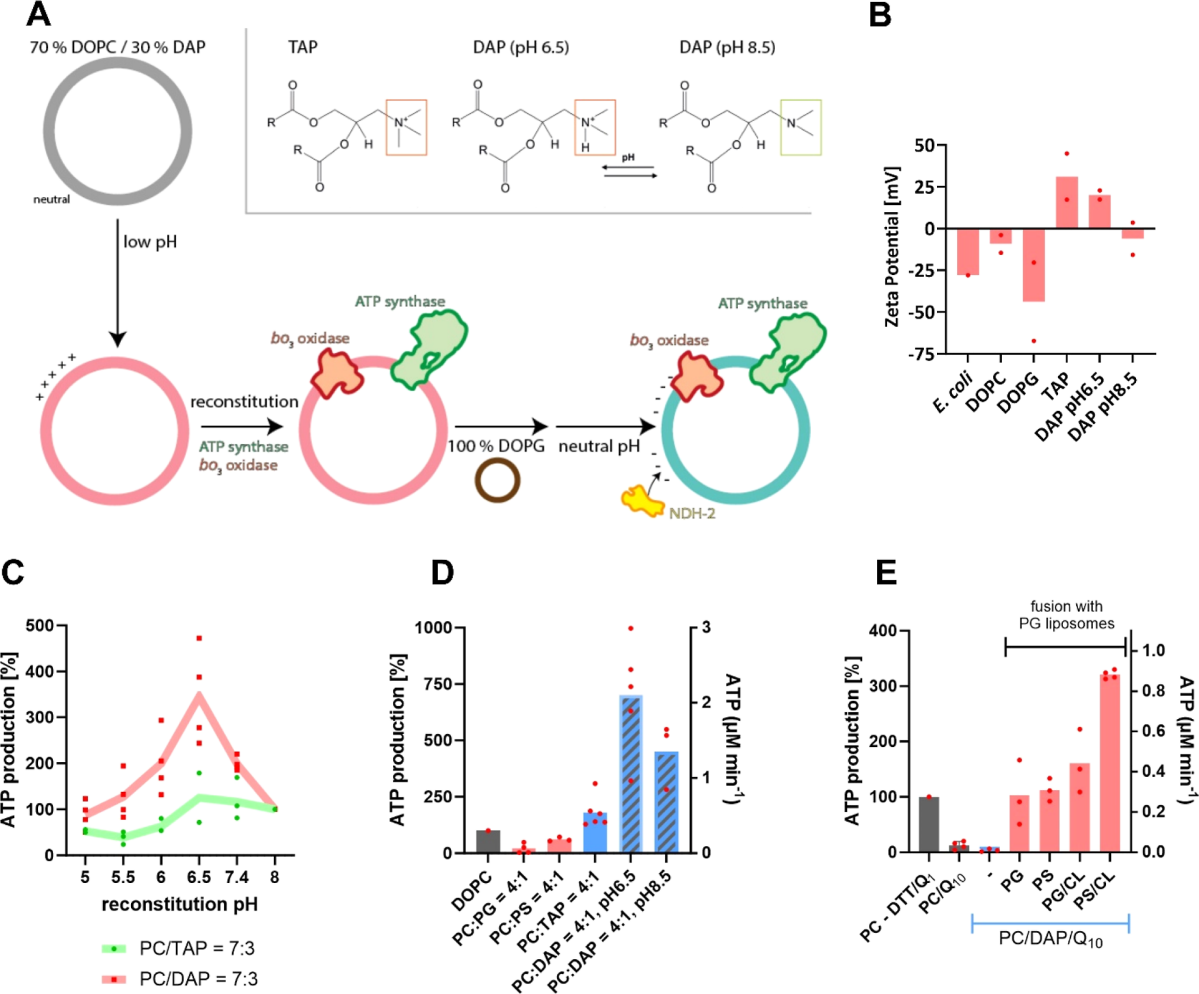
(A) Strategy to use the ionizable DODAP lipid to temporarily provide a positively charged membrane which becomes negatively charged upon liposome fusion and pH adjustment. Initially neutrally charged liposomes become temporarily positive when applying acidic pH, under which condition also coreconstitution (ATP synthase and *bo*_3_ oxidase) is performed. Subsequent lipid mixing with negatively charged liposomes (e.g., 100% PG, brown) and physiological pH renders the liposome membrane overall negative, thus allowing NDH-2 to interact. Headgroups of TAP and ionizable DAP lipids are depicted. (B) Zeta potential measurements of differently charged liposomes. (C) Comparison of ATP synthesis rates between permanently (DOTAP) and transiently (DODAP) positively charged liposomes at different pH values. ATP production was chemically initiated with Q_1_ and DTT. (D) Normalized ATP synthesis efficiency induced chemically (Q_1_/DTT) in uncharged (gray), negatively charged (red), permanently positively charged (light blue), or ionizable liposomes (PC/DAP, different reconstitution pH, blue-gray mesh). (E) Normalized ATP synthesis induced by NDH-2/NADH (except first column) using DOPC/DODAP liposomes fused with different negatively charged liposomes (see text for details).

This finding left us with a conundrum, as the positively charged lipids required for the desirable *bo*_3_ oxidase orientation are highly incompatible with the energization method via NDH-2 and membrane-embedded Q_8_, which require a negatively charged surface (see above). Are there alternative approaches to orient a membrane protein? We, along with others, recently described successful efforts modulating the orientation of proteorhodopsin by (post-) translationally attaching a large soluble unit that guides orientation,^33,35^ but excluded this method for the purpose here, given the large size of *bo*_3_ oxidase, its multisubunit organization, and its strong dependency on electrostatic interaction during reconstitution. Instead, we decided to modulate the surface charge between overall positive during reconstitution and overall negative during measurements performed with NDH-2. This is, however, not possible using DOTAP or EPC lipids that are permanently positively charged lipids independent of the environmental conditions. This is different in ionizable lipids, which carry a headgroup that adapts its charge to the surrounding pH value (Figure 4A).^36,37^ We reasoned that such pH-sensitive lipids could be used to transiently render liposomes positively charged at low pH to favorably orient the oxidase during the reconstitution process. Upon an increase of the pH value, the ionizable lipids would lose their positive charge and take on a neutral state, thereby eliminating the unfavorable impact of positive lipids on enzyme activity. To render the proteoliposomes negatively charged as required for the interaction with NDH-2, we planned to exploit membrane fusion between positively and negatively charged liposomes, as described by us and others.^7,34,36,38^ At low pH, the ionizable lipids will be positively charged and undergo rapid lipid mixing when in contact with negatively charged liposomes,^36^ leading to an overall neutral surface charge. Upon a change in the surrounding pH value, pH-sensitive lipids are deprotonated and lose their positive charge, resulting in net negatively charged liposomes. The overall strategy is depicted in Figure 4A. In this work, we have focused on the use of DODAP as an ionizable lipid (Figure 4A, inset), which has a secondary amine as a headgroup that can be protonated with a reported p*K*_a_ of ∼6.5.^36,37^ However, other ionizable lipids, such as DOBAQ or DODMA, might also be suitable but have not been included in this study.

We verified our hypothesis by assessing the surface properties of differently charged liposomes by using zeta potential measurements (Figure 4B). In line with our expectations, zwitterionic DOPC liposomes showed a zeta potential close to 0 mV. In contrast, the constantly negatively charged membranes (*E. coli* mimetic membrane and PC/PG = 4:1) showed a negative zeta potential, while the positively charged liposomes (PC/TAP = 4:1) showed a positive zeta potential. Finally, depending on the pH of the buffer, liposomes containing ionizable lipids (PC/DODAP = 4:1) showed a neutral or positive zeta potential at pH values of 8.5 or 6.5, respectively.

As an acidic pH is required to render DODAP positive during reconstitution, we first examined the compatibility of our coreconstitution method in the pH range from 5.0 to 8.5 in liposomes containing 70% DOPC and 30% DODAP. The same experiments were performed with liposomes containing 30% DOTAP, which remain positively charged across all pH values tested. ATP synthesis was measured in all samples, and the data was normalized to the ATP synthesis activity at pH 8.5 (Figure 4C). In DOTAP liposomes, ATP synthesis was approximately constant in the pH range between 8.0 and 6.5 before declining at lower pH values. The exact reason for this decline remains unknown, as the *E. coli* ATP synthase has been shown to withstand pH values as low as pH 5 without loss of function^39^ when reconstituted into liposomes. We therefore assume that either the activity or the reconstitution efficiency of the *bo*_3_ oxidase was affected at lower pH values. In DODAP liposomes, ATP synthesis already took place at pH 8.5 and increased further until pH 6.5 before declining at lower pH levels, similar to the low pH effect observed with DOTAP liposomes. In the following, the reconstitution pH was either 6.5 or 8.5, and no further optimization of the pH was performed.

Inspired by the result that coreconstitution in the presence of DODAP leads to improved orientation of the *bo*_3_ oxidase and thus a higher ATP synthesis rate, we strived to compare the rates of this method with our previously best performing liposomes consisting of 100% DOPC, as described by Nilsson et al.^6^ For comparability’s sake, a fixed number of 4 *bo*_3_ oxidases and 2 ATP synthases per 100 nm liposome (based on *bo*_3_ oxidase titration, Figure S5) was used in all following experiments. The electron donor and mediator pair DTT and Q_1_ were further used as a reducing system (Figure 4D). Using the ATP synthesis efficiency in 100% DOPC liposomes as a reference (Figure 4D, gray bar), lower values were observed in liposomes supplemented with 20% of negatively charged phospholipids (PG, PS). A roughly 2-fold better ATP production compared to DOPC liposomes was observed in the presence of 20% permanently positively charged lipid (DOTAP). This observation agrees well with the earlier experiments described in Figure 3H. Interestingly, in the presence of 20% DODAP lipids at a reconstitution pH 6.5, a ∼ 7-fold higher ATP synthesis rate was found compared to the 100% DOPC reference. If the reconstitution was performed at pH 8.5, the increase was still ∼4-fold over 100% DOPC. From these data, we can draw several conclusions. First, positively charged lipids such as DOTAP and DODAP at pH 6.5 indeed significantly increase the ATP synthesis rate by correctly orienting the *bo*_3_ oxidases. Second, the effect is stronger in DODAP lipids than that in DOTAP lipids. This indicates that the presence of permanently positively charged lipids reduces ATP synthesis efficiency, seeing how the DODAP lipids in their neutral state outperform DOTAP under ATP synthesis assay conditions (pH 7.4). Third, the effect is also observed in liposomes containing DODAP at pH 8.5, despite the lipid being expected to be preferentially in its neutral state. A possible explanation might be the absence of the negatively charged phosphate group in the DODAP structure, otherwise present in all other phospholipids. To summarize, the use of ionizable lipids maximizes ATP synthesis rates in a minimal oxidative phosphorylation system by promoting unidirectional orientation of the *bo*_3_ oxidase and thus more productively creating the *pmf*. We have tested the long-term stability of these liposomes at 4 °C and room temperature and found only a small loss of activity after 24 h, indicating that the presence of ionizable lipids does not affect protein activity (Figure S7).

For the final set of experiments, liposomes (PC/DAP = 4:1) containing 2% Q_10_ were used, and reconstitution was performed at pH 6.5 based on the results above. In earlier experiments, we had used bacterial ubiquinone Q_8_ but later switched to much more affordable Q_10_, which showed similar activities in our hands (Figure S6). The strategy was to fuse empty negatively charged liposomes immediately after reconstitution with the still positively charged proteoliposomes containing *bo*_3_ oxidase and ATP synthase. Rapid lipid mixing is expected to occur, rendering the surface overall negatively charged upon the subsequent neutralization of the buffer and deprotonation of the DODAP lipids. As negatively charged lipids, we prepared liposomes composed of either 100% PG or PS or a mixture of PG or PS with 30% cardiolipin (CL), a unique lipid present in bacteria and mitochondria that has been proposed to play an active role during oxidative phosphorylation.^40^ Fusion was initiated by the addition of an equimolar amount of negatively charged liposomes (100 nm diameter) to the reconstitution mixture 5 min prior to measurements. To energize *bo*_3_ oxidase proton pumping, ∼ 200 nM NDH-2 and ∼200 μM NADH were added. The reference with 100% DOPC was energized with DTT/Q_1_, as described above (first gray bar). The results of these experiments are displayed in Figure 4E.

No ATP production was observed in 100% DOPC (second gray bar) as well as in PC/DAP/Q_10_ liposomes that are both uncharged at the measured pH level, preventing NDH-2 activity and thus quinone reduction, in accordance with results shown in Figures 1C and 2C. Impressively, ATP synthesis comparable to the reference (100% DOPC, Q_1_/DTT) was observed when the same PC/DAP/Q_10_ liposomes were incubated with either 100% PG or PS liposomes before the measurement. This rate was even increased if liposomes containing cardiolipin were used, and experiments using PS/CL showed a reproducible 3-fold ATP synthesis rate over the reference value of 100% DOPC energized with DTT/Q_1_.^1,2^ These experiments convincingly show that the proposed procedure renders the final proteoliposome surface negative, enabling efficient NDH-2 activity on membrane-embedded ubiquinone Q_10_, leading to proton pumping of the oriented *bo*_3_ oxidase, *pmf* creation, and ATP synthesis. The difference in PG of PS is in agreement with earlier reports that PS promotes membrane fusion more strongly than PG,^41^ and the activating effect of cardiolipin suggests an active role of this special lipid. From our experiments, we cannot discriminate if full fusion occurred between the two types of liposomes or if only lipid mixing occurred in the outer leaflet. However, the two scenarios are not expected to make a functional difference, as only the outer membrane leaflet is implicated in NDH-2 binding via its two C-terminal helices.

### Concluding Remarks

Bottom-up assemblies of membrane proteins into synthetic vesicles with the purpose to artificially produce ATP have led to various innovations and strategies over the past few years.^1,42^ Our group has contributed with methodology to coreconstitute the terminal quinol oxidase *bo*_3_ and ATP synthase from *E. coli* and showed that this combination allows very high rates of ATP synthesis, surpassing the values of light-driven systems.^1^ In these experiments, redox-driven proton pumping was energized using a synthetic reductant DTT with a midpoint potential (−0.33 V) similar to that of NADH. However, unlike NADH, oxidized DTT is accumulated and cannot be regenerated by enzymatic reactions. In addition, DTT is unable to reduce membrane-embedded long-chain quinones, and instead synthetic short-chain quinones such as Q_1_, Q_2_, or decylubiquinone are used. Based on the same reconstitution procedures, Biner et al.^3^ overcame this limitation by coreconstitution of proton-pumping mammalian complex I and *E. coli* ATP synthase into liposomes containing ubiquinone Q_10_. The quinone pool was regenerated using nonproton-pumping alternative oxidase, a peripheral membrane protein from *Trypanosoma brucei*. Here, we expand our previous system of *bo*_3_ oxidase and ATP synthase using NDH-2 as an electron entry point for membrane-embedded quinone. To overcome the apparently incompatible prerequisites of positively charged liposomes for *bo*_3_ oxidase reconstitution and negatively charged liposomes for NDH-2 activity, we propose the use of the pH sensitive lipid DODAP, which is positively charged at low pH but uncharged at neutral or alkaline pH values. The final negative charge of the proteoliposomes is achieved via charge-mediated fusion with negatively charged liposomes. We find that the system strongly promotes uniform orientation of *bo*_3_ oxidase in the desired orientation, enabling ATP synthesis rates with water-soluble Q_1_ via DTT and membrane-embedded Q_10_ via NADH that surpass the previously best activities by 7-fold or 3-fold, respectively. The use of pH-sensitive lipids also suppresses the negative effects on enzyme functionality that are found in permanently positively charged lipids. The efficiency of the ATP-generating system could be further improved by replacing NDH-2 with complex I, reflecting the entire respiratory chain of *E. coli*. Peripheral membrane proteins like NDH-2 used here or alternative oxidase used in Biner et al.^3^ can be conveniently added to the system from the outside, but do not exploit the entire thermodynamical potential for *pmf* generation of the NADH to oxygen electron transfer reactions.

We conclude the discussion with a number of remarkable aspects in relation to the mechanism of enzyme reconstitution that have surfaced during this project.

First, data presented in this work was collected over many years, and countless preparations of ATP synthase and *bo*_3_ oxidase and many batches of synthetic lipids (mainly DOPC and PG, but also mixtures of PC, see methods) have been used. During this time, the suppressing effect of liposomes containing negatively charged lipids on ATP synthesis observed by Nilsson et al.^6^ was reproduced dozens of times. However, the extent of the effect varied somewhat unpredictably, albeit seemingly consistent within protein preparation. How could the quality of a protein preparation influence the orientation during reconstitution? A likely explanation is that the preparations vary in their content of copurified lipids, i.e., how complete their annular lipid layer has been removed by the detergent during the purification process. Our data clearly show that orientation is guided by the interaction of the protein with lipid head groups, and a more severely lipid-stripped enzyme is therefore expected to react stronger to the lipid composition of the liposomes, while an enzyme still fully embedded in its annular layer might be less affected. We consider the same effect also likely to be the explanation for the different impact of lipids on the activity of different NDH-2 batches. Our data show that lipids also stimulate NDH-2-mediated reduction of short-chain ubiquinones in detergent solution, suggesting that lipids affect electron transfer from FAD to the Q-site. As NDH-2 from *E. coli* is purified via membrane solubilization by detergents, strongly bound lipids could be copurified to different extents in NDH-2 preparations.

Second, we were surprised to see the strong impact of lipids on the orientation of *bo*_3_ oxidase, which explains a large portion of the effects described by Nilsson et al.^6^ An influence of surface charge on the orientation has been observed for proteorhodopsin, a small membrane protein with almost no extramembranous domains.^18^ For larger proteins, the presence of a large extramembranous domain has been considered the dominant effect. In that respect, it is interesting to note that the earliest *bo*_3_ oxidase structure obtained by Abramson et al.^43^ only showed a periplasmic extramembranous domain (PDB access: 1FFT), which favors the right-side-out orientation. If this is the dominant orientation, why do we then observe ATP synthesis at all when the majority of enzymes would pump toward the outside, outcompeting the inside-out vesicles that acidify the liposome lumen, enabling ATP synthesis? With the rise of high-resolution cryo-EM, new structures of the *bo*_3_ oxidase were published, also showing an extramembranous domain on the cytoplasmic side of the enzyme (e.g., PDB access: 7CUB, 7N9Z, and 8QQK).^44,45^ This cytoplasmic domain is somewhat smaller than the periplasmic one and exhibits a distinct belt of positive charges close to the membrane surface that is absent on the periplasmic side. Such a positive surface is in agreement with the positive inside rule for membrane proteins.^46−48^ Our data suggest that the presence of an extramembranous domain dominates the orientation in neutral lipids (e.g., DOPC) but that in the presence of a net surface charge, the positively charged belt becomes the dictating factor of the reconstitution outcome. In other words, in the case of a negative liposome surface, the positively charged belt on the cytoplasmic side of the protein contacts the liposomes first, yielding a predominantly right-side-out orientation. In the presence of positively charged lipids, however, the belt is repelled, and a predominantly inside-out orientation is obtained. The important role of such electrostatic interactions is corroborated if high salt conditions were applied, which weakened the effect.

Third, we found a considerable and highly reproducible impact on *bo*_3_ oxidase orientation if ATP synthase was coreconstituted. This is an intriguing and interesting finding that we cannot currently explain but would like to address in the future. The observation suggests that incorporation of enzymes into liposomes is not an individual process but might happen in a coordinated manner involving cooperativity effects. Assuming that ATP synthase reconstitution proceeds quickly, its interaction with the membrane could produce local membrane disturbances that could act as preferred entry points for further protein insertions. As ATP synthase orients independently of the lipid composition,^6^ its presence in the inside-out orientation might stimulate incorporation of *bo*_3_ oxidase that is correct relative to the orientation found in nature, i.e., also in the inside-out orientation enabling formation of *pmf*, as it is observed. Hints for nonhomologous processes during reconstitution were recently shown by Veit et al.^49^ quantifying enzyme incorporation on a single vesicle level. They found that ∼50% of liposomes did not contain any protein despite a sufficiently high protein to liposome ratio that, according to a Poisson distribution, should not render any empty liposomes. Further studies are required to understand these processes in more detail. The discussed points reinforce the notion that membrane protein insertion into lipid bilayers is a complex process that is affected by several parameters.

## Materials and Methods

Chemicals, if not otherwise stated, were purchased from Sigma-Aldrich.

### Expression and Purification of bo_3_ Oxidase Wildtype and Mutants

Single-cysteine *bo*_3_ oxidase mutants (ID578C, IIIA21C) were constructed from plasmid pETcyoII,^50^ encoding for the entire *cyo* operon. Wildtype and mutant *bo*_3_ oxidase were expressed in *E. coli* strain C43Δcyo^51^ cells. Cells were grown either in M63 minimal medium (3 g/L KH_2_PO_4_, 7 g/L K_2_HPO_4_, 0.5 mg/L FeSO_4_, 100 μg/mL ampicillin, 1 mM MgSO_4_, 100 mg/L thiamine, 10 μM CuSO_4_, 0.2% glucose, and 0.2% NH_4_Cl) or in petcyo medium (0.5% yeast extract, 1% peptone from meat, 1% NaCl, 0.5% glycerol, 2 mM MgSO_4_, 30 μM FeSO_4_, and 10 μM CuSO_4_) containing 100–200 μg/mL ampicillin in a LEX48 system at 38 °C. Expression was induced at an OD600 of 0.5–1 with 1 mM IPTG (Santa Cruz) followed by an additional incubation at 38 °C for at least 4–5 h. Cells were harvested by centrifugation, resuspended in Buffer D (50 mM HEPES pH 8.3, 5 mM MgCl_2_) containing DNase I and protease inhibitors PMSF (1 mM) and Pefabloc (spatula tip; Biomol), and lysed by 3-4 passes through MAXIMATOR (HPL6 high-pressure homogenizer, maximator AG) at 2 °C. After cell debris was removed by centrifugation (8,000*g*, 0.5 h, 4 °C), membranes were harvested by ultracentrifugation (200,000*g*, 1 h, 4 °C) and resuspended in Buffer E (50 mM K_2_HPO_4_, pH 8.3) containing 5 mM imidazole. Solubilization was performed with 1% DDM (glycon biochemicals GmbH) for 2 h at 4 °C (typically with additional PMSF), followed by ultracentrifugation (200,000*g*, 45 min, 4 °C). Solubilized protein was loaded on prepacked 5 mL HisTrap columns (GE Healthcare), washed with buffer E containing 0.05% DDM and 35 mM imidazole, and eluted with the same buffer containing 100 mM imidazole. Fractions containing *bo*_3_ oxidase were pooled and concentrated with a 100 kDa MWCO Amicon Ultra-15 filter (Merck Millipore). The pooled fraction was divided into aliquots, frozen in LN_2_, and stored at −80 °C.

### Expression and Purification of ATP Synthase in Buffer S

F_1_Fo-ATP synthase from *E. coli* was expressed as described in^33^ using plasmid pBWU13 β-His and *E. coli* DK8 cells.^30^ Cells were harvested by centrifugation and broken by 3 passes through MAXIMATOR (HPL6 high-pressure homogenizer, Maximator AG) at 1200 bar at 2 °C in Buffer A (50 mM HEPES pH 8.0, 100 mM NaCl, 5% glycerol) containing D Nase I (spatula tip) and protease inhibitors PMSF (0.1 mM) and Pefabloc (spatula tip; Biomol). After removal of cell debris (centrifugation at 5,000*g* for 0.5 h, 4 °C), membranes were pelleted by ultracentrifugation (175,000*g*, 1.5 h, 4 °C) and resuspended in 10 mM Tris–HCl pH 7.5 (1 mL per g of wet cells). For solubilization, homogenized membranes were diluted with 2 x solubilization buffer S (50 mM HEPES pH 7.5, 100 mM KCl, 250 mM sucrose, 20 mM imidazole, 40 mM 6-aminohexanoic acid, 15 mM *P*-aminobenzamidine, 5 mM MgSO_4_, 0.1 mM Na_2_-EDTA, 0.2 mM DTT, 0.8% soy bean type II asolectin, 1.5% *n*-octyl β-*d*-glucopyranoside, 0.5% sodium deoxycholate, 0.5% sodium cholate, and 2.5% glycerol; Magic buffer) in a ratio of 1:1 and incubated at 4 °C for 1.5 h while stirring. Nonsolubilized material was removed by ultracentrifugation (200,000*g*, 30 min, 4 °C), and the supernatant was looped on a prepacked 5 mL HisTrap column (GE Healthcare) equilibrated with buffer *S* at 4 °C for 2 h. The column was washed with 5 column volumes (cv) of buffer *S* containing 40 mM imidazole and 3 cv of buffer *S* containing 90 mM imidazole. Purified protein was eluted with buffer *S* containing 250 mM imidazole, and fractions containing ATP synthase were identified by ATP regenerating assay^52^ and pooled. The pooled fraction was divided into aliquots without concentrating, frozen in LN_2_, and stored at −80 °C.

### Purification of ATP Synthase in LMNG

Cells were harvested, and membranes were prepared, as described above, in Buffer B (50 mM MOPS/NaOH pH 8.0, 100 mM NaCl, 5 mM MgCl_2_, and 5% glycerol). Pelleted membranes were resuspended (2 mL per g of wet cells) in Buffer C (50 mM MOPS/NaOH pH 8.0, 100 mM NaCl, 5 mM MgCl_2_, 30 g/L sucrose, and 10% glycerol). For solubilization, LMNG (Anatrace) was added to a final concentration of 2% from a 5% stock solution (in water). After the suspension was stirred for 30 min at room temperature and 30 min at 4 °C in the presence of 1 mM PMSF, 5 mL of Buffer C was added per g of membranes, and nonsolubilized material was removed by ultracentrifugation (200,000*g*, 0.5 h, 4 °C). The supernatant was loaded onto a prepacked 5 mL HisTrap column (GE Healthcare) in the presence of 10 mM imidazole via loop-loading for 2 h at 4 °C. Bound protein was eluted via gradient elution from 20 to 400 mM imidazole in Buffer C containing 0.005% LMNG. Fractions containing ATP synthase were identified by the ATP regenerating assay,^52^ pooled, and concentrated with a 100 kDa MWCO Amicon Ultra-15 filter (Merck Millipore). The pooled fraction was divided into aliquots, frozen in LN_2_, and stored at −80 °C.

### Expression and Purification of NDH-2

NDH-2 was expressed in BL21Δcyo(DE3) or BL21(DE3)pLysS using plasmid pETNDH-2_N5 (gift from Robert Gennis from the University of Illinois). Cells were grown in LB medium containing 100 μg/mL ampicillin and 1 mM MgSO_4_ either in a shaker or in a LEX48 system at 37 °C until OD600 reached 0.6, followed by induction with 0.5–1 mM IPTG. NDH-2 was expressed for an additional 4 h at 37 °C and cells were harvested by centrifugation and resuspended in Buffer F (10 mM HEPES pH 7.4, 100 mM NaCl, and 10 mM KCl) containing 20% glycerol, 2 mM MgCl_2_, 1 mM PMSF, and a spatula tip of Pefabloc (Biomol) and D Nase I. Cells were broken by 3 passes through MAXIMATOR (HPL6 high-pressure homogenizer, Maximator AG) at 1500–2000 bar at 2 °C and unbroken cells were removed by centrifugation (8,000*g*, 30 min, 4 °C) before membranes were pelleted by ultracentrifugation (175,000*g*, 1 h, 4 °C). Membranes were resuspended in Buffer F (2 mL/g of wet cells) containing 1 mM PMSF and a Pefabloc spatula tip of Pefabloc. For solubilization, 2% DDM (glycon biochemicals GmbH) was added from a 20% stock solution (in water), and the sample was diluted with Buffer F to a final DDM concentration of 1%. After incubation for 1 h at 4 °C while stirring, nonsolubilized material was removed by ultracentrifugation (175,000*g*, 1 h, 4 °C), and 10 mM imidazole was added to the supernatant. The supernatant was then bound either onto a prepacked 5 mL HisTrap column (GE Healthcare) or Ni-NTA beads equilibrated with Buffer F containing 0.05% DDM and 10 mM imidazole. Bound protein was eluted either via gradient elution in Buffer F containing 0.05% DDM from 5 to 300 mM imidazole or washed first with 10 cv of Buffer F containing 0.05% DDM and 20 mM imidazole, followed by the same buffer containing 50 mM imidazole, and eluted with 5 cv of the same buffer containing 200 mM imidazole. Yellow or peak fractions were pooled and concentrated with a 100 kDa MWCO Amicon Ultra-15 filter (Merck Millipore). The pooled fraction was divided into aliquots, frozen in LN_2_, and stored at −80 °C.

### Expression and Purification of Fumarate Reductase

Fumarate reductase from *E. coli* was overexpressed and purified, as described.^24^

### Site-Specific Labeling with DY647P1-Maleimide

Labeling was performed as described.^21,53^ In brief, purified single-cysteine mutants were diluted with maleimide reaction buffer (20 mM HEPES pH 6.5, 100 mM KOAc, and 0.05% DDM) in a ratio of 1:5 to adjust the pH. The cysteines were reduced with 0.4 mM TCEP, and the samples were incubated with a 10-fold excess of DY647P1-maleimide (Dyomics GmbH) over the protein overnight at 4 °C (end-overtail rotation). Excess dye was removed by gel filtration (CentriPure P10 or P50, emp Biotech GmbH) using maleimide reaction buffer for equilibration and elution and three cycles of diluting and concentrating with a 100 kDa Amicon Ultra-15 filter (Merck Millipore).

### Liposome Preparation

Lipids used were purchased from avanti polar lipids (18:1 cardiolipin; *E. coli* extract polar; 18:1 (Δ9-Cis) PC (DOPC); 18:1 (Δ9-Cis) PE (DOPE); 18:1 TAP (DOTAP); and 18:1 DAP (DODAP)) or lipoid (LIPOID E PC S; LIPOID PG 18:1/18:1; and LIPOID E PE). Lipids were dissolved in chloroform and mixed in appropriate ratios. If necessary, 2 mol % Q_8_ or Q_10_ (dissolved in chloroform) was mixed with lipids. Chloroform was evaporated in a desiccator overnight, and lipids were resuspended either in Buffer L1A (20 mM HEPES pH 7.5, 2.5 mM MgCl_2_, and 50 g/L sucrose) or L1B (50 mM MOPS-BTP pH 6.75) at a concentration of 5–10 mg/mL for coupled ATP synthesis measurements, in Buffer L2 (20 mM HEPES pH 7.4, 200 mM NaCl, and 20 mM KCl) at a concentration of 5–10 mg/mL for NADH oxidation measurements with NDH-2, or in Buffer L1B (50 mM MOPS-BTP pH 6.75) at a concentration of 40 mg/mL for orientation measurements. To get unilamellar liposomes, the suspension was subjected to 7 cycles of freezing (liquid nitrogen) and thawing (at 29.4 °C), each cycle followed by vortexing for some seconds. Liposomes were divided into aliquots, frozen in LN_2_, and stored at −80 °C.

Liposomes used for coupled ATP synthesis measurements were thawed directly before use and extruded 21 times through a Whatman polycarbonate membrane (Little Chalfont or Sigma-Aldrich) with a 100 nm pore size.

Liposomes used for NADH/ubiquinone oxidoreductase measurements of NDH-2 were thawed directly before use and sonicated with a tip sonicator (5 min, pulse on 30 s, pulse off 30 s, amplitude 40%).

Liposomes used for orientation measurements were thawed directly before use, diluted with Buffer L1B to 10 mg/mL, and extruded 21 times through a Whatman polycarbonate membrane (Sigma-Aldrich) with a 100 nm pore size.

### Reconstitution/Co-Reconstitution of Membrane Proteins

Reconstitution of the individual enzymes ATP synthase or *bo*_3_ oxidase was performed similarly to the coreconstitution of the two enzymes, as described by von Ballmoos et al.^2^ Briefly, liposomes were partially solubilized with 0.4% sodium cholate using a 30% stock solution (in water) before the enzymes were added. For coupled ATP synthesis measurements, we used 5 enzymes per liposome each (which usually was calculated as a theoretical mean value from measured enzyme concentration and calculated liposome concentration), while for orientation measurements, varying amounts of fluorescently labeled protein were used (3-5 enzymes per liposome) to adjust for fluorescence signal. The mixture was incubated for 0.5 h at 4 °C (let it stand) or at room temperature (300 rpm), followed by gel filtration (CentriPure P10 column, emp Biotech GmbH) to remove the detergent. Equilibration and elution were done either with Buffer R1 (20 mM HEPES, pH 7.5, 2.5 mM MgCl_2_, and 25 g/L sucrose) for coupled ATP synthesis measurements or with Buffer R2 (100 mM MOPS, pH 7.5, 25 mM K_2_SO_4_, 1 mM MgCl_2_) for orientation measurements. Depending on the downstream application, the liposomes were either pelleted by ultracentrifugation (type 70.1 Ti rotor, 200,000*g*, 1 h, 4 °C) or directly used for measurements.

For coreconstitution of *bo*_3_ oxidase, ATP synthase, and FRD, 250 μL of 5 mg/mL liposomes was partially solubilized with 0.6% cholate and mixed with the three enzymes before the detergent was removed by gel filtration (CentriPure P10 column, emp Biotech GmbH) after 30 min.

### Coupled ATP Synthesis Activity Measurements

Coupled ATP synthesis activity was measured as described.^6^ For DTT/Q_1_-induced ATP synthesis, briefly, proteoliposomes were mixed with 500 μL of measuring buffer M (20 mM Tris-PO_4_ pH 7.5, 5 mM MgCl_2_, 4 mM DTT, 80 μM ADP, and 0.2 mg/mL ATP Bioluminescence Assay Kit CLS II (Roche)). After a baseline was measured with a GloMax 20/20 Luminometer (Promega) for 30 s, the reaction was started with 20 μM Q_1_, and luminescence was measured for 90 s. The reaction was stopped with the addition of at least 50 μM KCN. A known amount of ATP was added, and ATP synthesis rates [pmol ATP/s] were calculated by subtraction of the baseline slope and normalization with ATP addition.

For NDH-2/NADH-induced or FRD-induced coupled ATP synthesis activity measurements, proteoliposomes containing Q_8_ or Q_10_ were mixed with buffer M lacking DTT, and baseline luminescence was detected. The reaction was started by adding either 200 μM NADH or 300–500 nM NDH-2 or 1 mM succinate, respectively. FRD was inhibited with 400 μM malonate.

K_M_ measurements shown in Figure S1A–D were performed as follows: The quinol *bo*_3_ oxidase, FRD, and ATP synthase were coreconstituted into liposomes formed from *E. coli* polar lipid extract (5 mg/mL, 100 nm) containing 2 mol % ubiquinone Q_8_. NDH-2 was added directly before the measurements, and ATP production was detected in a luminometer. (A) Succinate. The succinate concentration varied between 1 and 2000 μM, yielding an apparent *K*_M_ of 62.2 μM. (B) Ubiquinone Q8. The ubiquinone concentration was titrated from 0 to 3 mol % Q_8_, yielding a *K*_M_ of 1.0 mol %. (C) NADH. The NADH concentration was raised from 5 to 200 μM. The apparent *K*_M_ was 18.5 μM. (D) NDH-2. Purified *E. coli* NDH-2 was added for a final concentration between 6 and 600 nM. The observed *K*_M_ was 45.6 nM.

### NADH Oxidation Measurements with NDH-2

NADH/Q_2_ oxidoreductase activity of NDH-2 was measured spectroscopically at 340 nm with a Cary 60 UV–vis Spectrophotometer (Agilent Technologies). Absorption of 1 mL Buffer L2 (20 mM HEPES pH 7.4, 200 mM NaCl, 20 mM KCl) containing 100 μM NADH, 100 μM Q_2_ (homemade), 1 mg/mL liposomes, and 3.2–32 nM wildtype *bo*_3_ oxidase was measured until reaching a stable baseline, before NADH oxidation was initiated by addition of 0.8–80 nM NDH-2. NDH-2 activity was determined with the slope after NDH-2 addition and depicted as relative activity normalized to activity in the presence of ECPE liposomes.

### Orientation Determination

For the TCEP-based orientation determination assay, site-specifically DY647P1-labeled single-cysteine mutants were reconstituted into liposomes, as described above. After ultracentrifugation, liposomes were resuspended in Buffer R2 (typically 1/2 of the volume of liposomes used for reconstitution), and 10–100 μL were diluted in 1.4 mL 250 mM Tris–HCl pH 8.5. Fluorescence of DY647P1 was monitored (excitation 649 nm, emission 672 nm; slits 5/10 nm) on a cary eclipse fluorescence spectrometer (Agilent Technologies). After reaching a stable baseline (1 min), a first quenching plateau was induced by the addition of 14 mM tris(2-carboxyethyl)phosphine (TCEP). After 2.5 min, liposomes were solubilized by adding 0.05% Triton X-100 (20% stock solution, in water), leading to a total quench, and fluorescence was monitored until the signal was stable (5 min). The orientation was calculated as the ratio between the first and total quench.

### Zeta Potential Measurement

Proteoliposomes (10 mg/mL) harboring ATP synthase and *bo*_3_ oxidase (2:4 ratio; number of enzymes per vesicle) were used for zeta potential measurements performed with a Litesizer 500 instrument (Anton Paar, Austria) in an Omega cuvette (Anton Paar, Austria).

### Charge-Mediated Fusion with Proteoliposomes

Fusion buffer (20 mM HEPES) was prepared at desired pH levels with 4 x higher buffering capacity compared to the reconstitution buffer (5 mM). ATP synthase and *bo*_3_ oxidase were coreconstituted into DOPC/DOTAP/DODAP liposomes (ratio as indicated), as per previously described. The standard fusion approach was performed by adding X μL of proteoliposomes harboring ATP synthase and *bo*_3_ oxidase and X μL empty liposomes of negative charge (DOPG/DOPS/CL) to Y μL of fusion buffer, where *Y* = *X* + *X*. Fusion was performed for 5 min at 23 °C on a heat block while shaking (1,000 rpm). Typically, 20 μL of each proteoliposome species was fused in 40 μL of fusion buffer. Also, 4 *bo*_3_ oxidases and 2 ATP synthases per vesicle were used for measurements. NDH-2-driven ATP production was initiated with 200 nM NDH-2. Coupled reactions resulting in ATP synthesis were then followed by a luciferin/luciferase-based assay detected by the GloMax 20/20 Luminometer (Promega) according to the previous description.

## Supporting Information Available

Results showing titrations of synthetic respiratory chain components; labeling specificity; impact of coreconstitution on enzyme orientation; influence of positively charged lipids on coupled ATP synthesis; ATP synthesis with varying amounts of *bo*_3_ oxidase while having constant ATP synthase; stability measurements; and comparison of Q_8_ and Q_10_ as an electron mediator in the presented system (PDF)
