# Supplementary material for: Modulating Liposome Surface Charge for Maximized ATP Regeneration in Synthetic Nanovesicles
Source: ACS Synth Biol. 2024 Nov 26;13(12):4061–73. doi: 10.1021/acssynbio.4c00487 (PMC11669383; doi:10.1021/acssynbio.4c00487)
Supplement: Supplementary file 1 — sb4c00487_si_001.pdf [file sb4c00487_si_001.pdf]

Supplementary material

Modulating Liposome Surface Charge for maximized ATP Regeneration in Synthetic Nanovesicles

Sabina Deutschmann[1,2], Stefan Theodore Täuber[1&], Lukas Rimle[1,2&], Olivier Biner[1,2], Martin Schori[1], Ana-Marija Stanic[1] and Christoph von Ballmoos[1*]

1 Department of Chemistry, Biochemistry and Pharmaceutical Sciences, University of Bern, Freiestrasse 3, 3012 Bern, Switzerland

2 Graduate School for Cellular and Biomedical Sciences, University of Bern, Bern, Switzerland

& = contributed equally; *Corresponding author: christoph.vonballmoos@unibe.ch

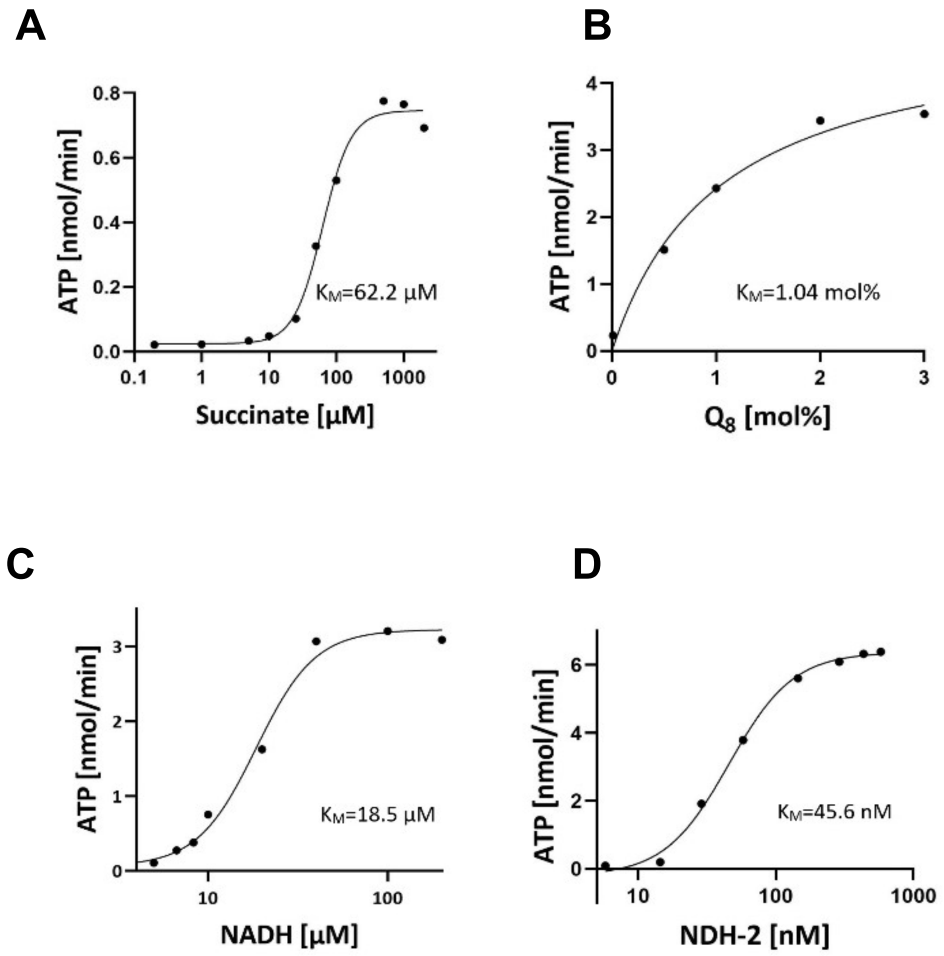

Figure S1. Titrations of synthetic respiratory chain components. *bo*$_3$ oxidase, ATP synthase and FRD were co-reconstituted into 5 mg/mL ECPE liposomes (100 nm), while NDH-2 was added to liposomes during measurements. ATP production was monitored with luciferin/luciferase. One component was titrated at a time, while the others were kept in excess (500 nM NDH-2; 200 µM NADH; 1 mM succinate; 2 mol % Q_8). Values were fitted in GraphPad Prism (Michaelis-Menten (Q_8); Sigmoidal, 4PL, X is concentration (Succinate, NADH, NDH-2)). KM values are indicated. A) Succinate dependency. B) Q_8 titration. C) NADH titration. D) NDH-2 dependency.

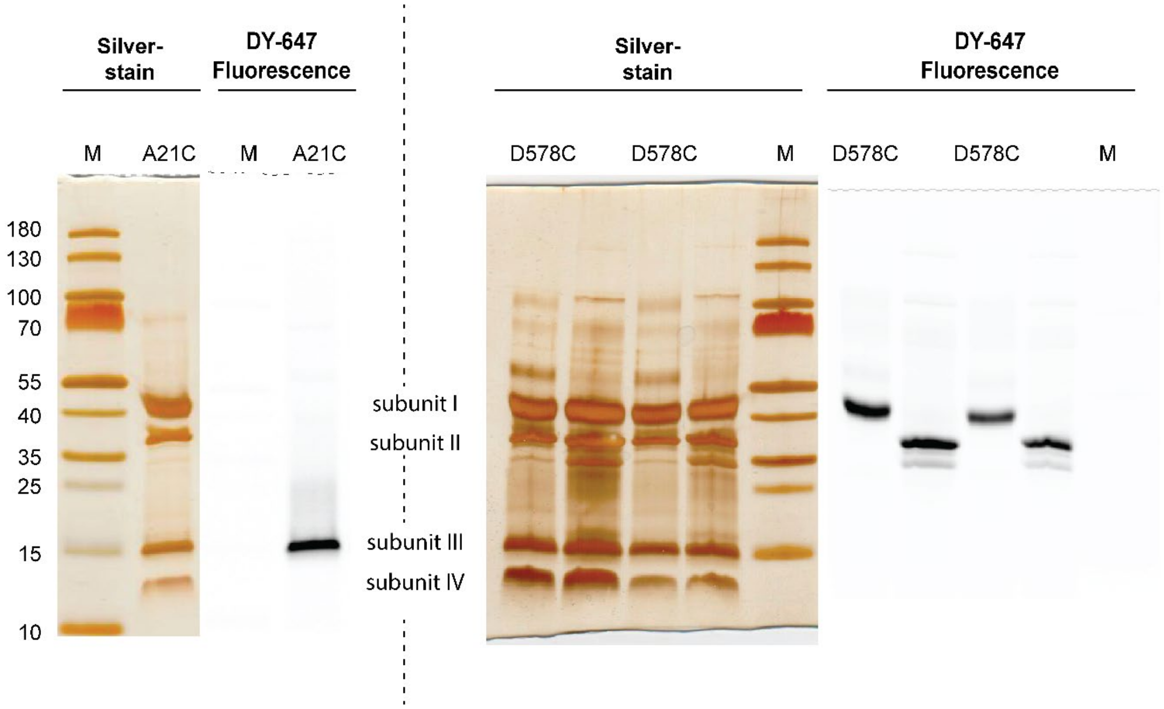

Figure S2. SDS PAGE and fluorescence scanning from single-cysteine *bo*3 oxidase-DY647P1 mutants IIIA21C (left) and ID578C (right). The additional two lanes in the gel on the right (not labelled) show subunit II labelling from variants that have not been used in this project but were included to present the Marker on the same gel as the D578C variants. Marker used: Page Ruler prestained protein ladder, 10-180 kDa, Thermo Fisher.

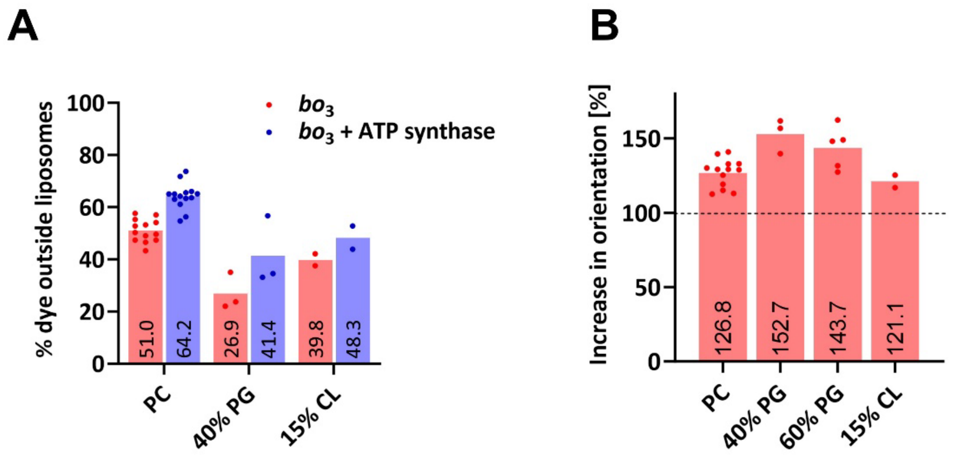

Figure S3. Comparison of *bo*3 orientation during reconstitution in the presence (blue) or absence (red) of ATP synthase**.** A) *bo*3 oxidase alone or together with ATP synthase was reconstituted in liposomes (10 mg/mL) partially solubilized with 0.4 % sodium cholate. After removal of sodium cholate by gel filtration, liposomes were pelleted by ultracentrifugation and orientation was determined via TCEP-based assay. B) Data from A) are represented as increase of *bo*3 oxidase in the inside-out orientation in the presence of ATP synthase compared to *bo*3 oxidase alone. The data of *bo*3 oxidase alone (red) are experiments independent of the results shown in figure 3C.

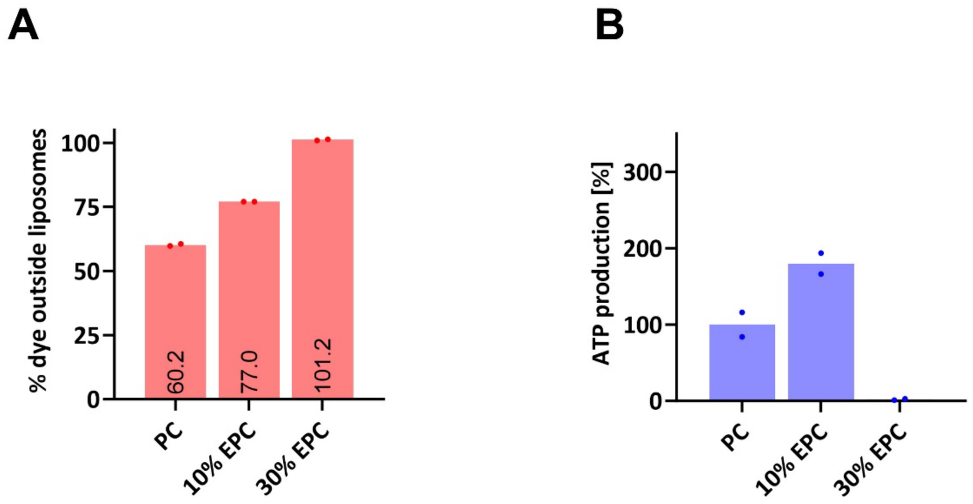

Figure S4. A) Orientation determination of *bo*$_3$ oxidase in liposomes consisting of a variable EPC content. B) ATP synthesis measured with proteoliposomes harboring *bo*$_3$ oxidase and ATP synthase consisting of 10 % and 30 % EPC and PC only.

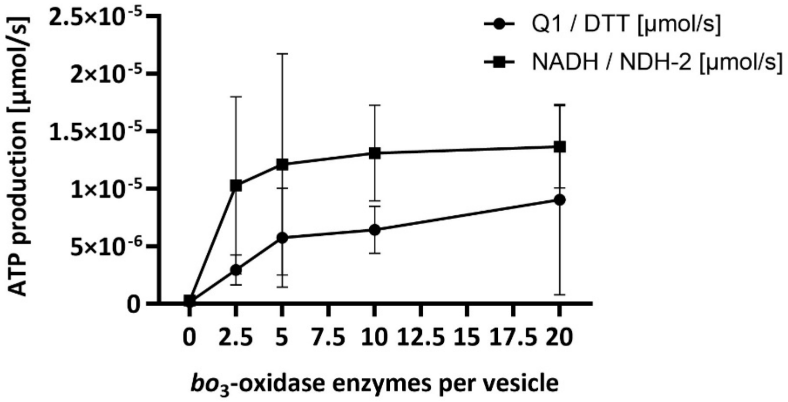

Figure S5. Titration of *bo*$_3$ oxidase when reconstituted together with ATP synthase. The number of *bo*$_3$ oxidase enzymes was varied from 0 to 20 enzymes per vesicle, while the number of ATP synthase per vesicle was kept constant (5 enzymes per vesicle). ATP production was monitored with luciferin/luciferase. The ATP production rate was used to identify the most economical combination with maximal ATP synthesis rates and minimal *bo*$_3$ oxidase input. Maximal ATP synthesis rates were obtained with 4 *bo*$_3$ oxidases per vesicle. ATP synthesis was either initiated with Q_1/DTT (circles) or with NDH-2/NADH (cubes).

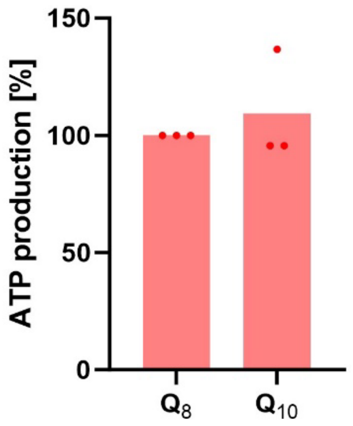

Figure S6. Comparison of membrane-embedded electron-mediators Q_8 and Q_{10} used for ATP synthesis initiated with NDH-2. Bacterial Q_8 (activity set to 100 %) shows similar activity as the more affordable Q_{10}.

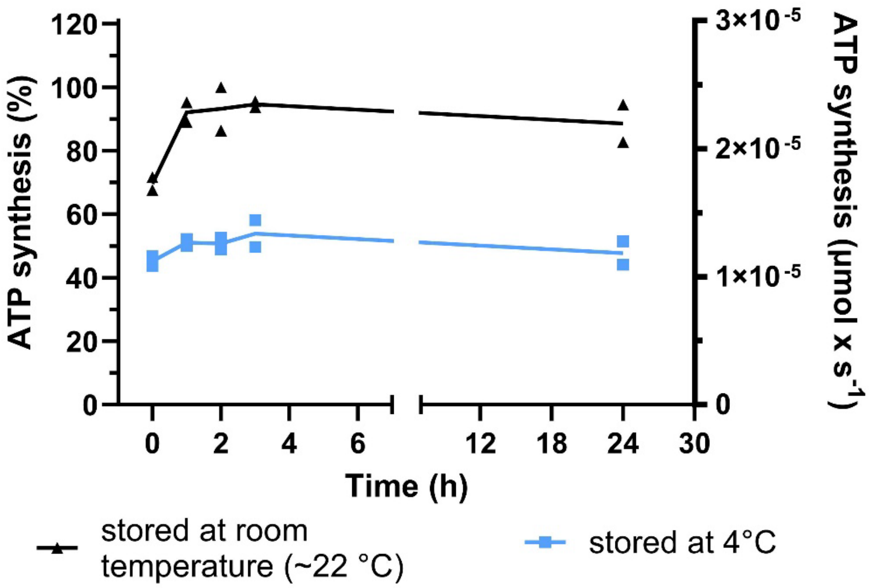

Figure S7. Stability of the proteoliposomes produced using ionisable lipid DODAP. Two independent reconstitutions were prepared in parallel and shown are the values of the measurements and a line connecting the mean values. Liposomes were kept at 4°C in an ice box or at room temperature. At the indicated times points, liposomes were mixed with assay buffer as described and ATP synthesis rates were determined using 2 mM DTT and 10 µM Q_1 as electron source or mediator, respectively.

Supplementary table 1:

Typical specific activities and concentration of purified proteins

	**NDH-2**	**FRD**	***bo*$_3$ oxidase**	**ATP synthase**
**Conc (µM)**	67 µM	15 µM	19.4 uM	7 µM
**Spec. activity**	45 s^{-1} (NADH oxidation)	11 s^{-1} (quinone reduction)	134.7 $e^- s^{-1}$	2.85 U/mg (ATP hydrolysis)
